# Efficacy of stereotactic body radiation therapy for locoregional recurrent pancreatic cancer after radical resection

**DOI:** 10.3389/fonc.2022.925043

**Published:** 2022-07-22

**Authors:** Xiaoqin Ji, Bin Zhou, Wei Ding, Jiasheng Wang, Wanrong Jiang, Yikun Li, Jun Hu, Xiangdong Sun

**Affiliations:** Department of Radiation Oncology, Affiliated Jinling Hospital, Medical School of Nanjing University, Nanjing, China

**Keywords:** SBRT, recurrent pancreatic cancer, NLR, surgery, pain

## Abstract

**Objective:**

This study aimed to analyze the efficacy and toxicity of stereotactic body radiotherapy (SBRT) for locoregional recurrent pancreatic cancer after radical resection.

**Methods:**

Patients with locoregional recurrent pancreatic cancer after surgery treated with SBRT in our institution were retrospectively investigated from January 2010 to January 2020. Absolute neutrophil-to-lymphocyte ratio (NLR) and platelet-to-lymphocyte ratio (PLR) recorded at pretreatment were analyzed. Endpoints included overall survival (OS), progression-free survival (PFS) and cumulative incidences of local failure (LF) and metastatic failure (MF).

**Results:**

A total of 22 patients received SBRT with a median prescribed dose of 40 Gy (range of 30-50 Gy)/4 to 7 fractions. The median OS of all patients was 13.6 months (95% CI, 9.6-17.5 months). 0-1 performance status (HR 12.10, 95% CI 2.04-71.81, P=0.006) and ≤2.1 pre-SBRT NLR (HR 4.05, 95% CI 1.21-13.59, P=0.023) were significant predictors of higher OS on multivariable analysis. The median progression-free survival (PFS) of the cohort was 7.5 months (95% CI, 6.5-8.5 months). The median time to LF and MF were 15.6 months and 6.4 months, respectively. The rate of MF as a first event was higher than that of first event LF. Pain relief was observed in all patients (100%) 6 weeks after SBRT. In terms of acute toxicity, grade 1 including fatigue (6, 27.3%), anorexia (6, 27.3%), nausea (4, 18.2%) and leukopenia (4, 18.2%) was often observed. No acute toxicity of grade 4 or 5 was observed. In terms of late toxicity, no treatment-related toxicity was found during follow-up.

**Conclusion:**

This study showed that SBRT can significantly reduce pain, effectively control local tumor progression, and have acceptable toxicity for patients with locoregional recurrence after radical resection of primary pancreatic cancer. Good performance status and lower pre-SBRT NLR were associated with improved overall survival.

## Introduction

Pancreatic cancer is a deadly disease with a 5-year survival rate of less than 9% (1). Surgical resection is the standard of care. However, surgical resection rate is only 20% (2). Even after radical resection, most patients will still have local and/or metastatic recurrence within 2 years (3, 4), and the 5-year survival rate is only 20-25% (5). One retrospective observational study reported that the recurrence rates of local, metastatic and synchronous local/metastatic pancreatic cancer were 17%, 60% and 23%, respectively (6). Symptoms of local recurrence include pain, bowel obstruction, portal hypertension, biliary obstruction, and malnutrition, which severely affect patients’ quality of life (7). However, successful treatments with adequate evidence for such recurrences of pancreatic cancer have yet to be established. Curative re-resection might result in a survival benefit. However, in most cases, reoperation is not attainable due to simultaneous distant metastases, vascular involvement, or poor physical condition (8–10). For such unresectable recurrences, conventional radiotherapy with or without chemotherapy can be considered. However, conventional radiotherapy is limited due to the presence of critical normal structures, such as small bowel, kidneys, and spinal cord, and further radiation can lead to unacceptable toxicity.

Recently, stereotactic body radiation therapy (SBRT) has attracted much attention. SBRT has unique advantages: (1) It can deliver high dose accurately to the target area with rapid dose fall off; (2) It enables a higher biological effective dose (BED) and (3) lower toxicity; (4) It enables the real-time tracking; (5) It has less treatment time (11–13). Thus, SBRT has gradually become an attractive radiation therapy technology, and been widely used in the treatment of pancreatic cancer (14, 15). SBRT combined with chemotherapy for the treatment of locally advanced pancreatic cancer can effectively control local tumors and alleviate local symptoms, prolong the overall survival of patients, and has tolerable toxicity (16–19).

Many studies have indicated that systemic inflammation and nutritional status, such as the systemic inflammation response index (SIRI), neutrophil to lymphocyte ratio (NLR), platelet-to lymphocyte-ratio (PLR) and prognostic nutritional index (PNI), were associated with the survival of many malignancies (20–23), including pancreatic cancer (24–27). These markers are promising predictors of clinical prognosis of cancer because they are inexpensive and easy to estimate. For example, the elevated baseline lactate dehydrogenase (LDH) level and derived NLR were associated with poor survival regardless of treatment modality for patients with metastatic non–small cell lung cancer (28).

However, there are few studies on the role of serum inflammation-based and/or nutritional markers in predicting the prognosis of locoregional recurrent pancreatic cancer after SBRT. Therefore, this study evaluated the efficacy and safety of SBRT in the treatment of patients with locoregional recurrent pancreatic cancer after radical surgery.

## Methods

### Patients

This is a retrospective study of 22 patients with locoregional recurrent pancreatic cancer after surgery. They underwent SBRT from January 2010 to January 2020 in Jinling Hospital. Patient inclusion criteria were as follows: (1) histologically confirmed as pancreatic adenocarcinoma; (2) R0 resection; (3) Comprehensive clinical and imaging examinations (including computed tomography (CT), magnetic resonance imaging (MRI) and/or 18-fluorodeoxyglucose positron emission tomography/computed tomography (18F-FDG-PET/CT)) before treatment confirmed the presence of recurrent lesions after surgery; (4) Considered surgically inoperable; (5) All patients’ medical records and radiotherapy documents had been carefully reviewed. Exclusion criteria were as follows: (1) prior history of malignant tumors in other areas; (2) prior in-field radiotherapy. Blood samples were routinely collected within 1 week before SBRT. The NLR was calculated as the number of neutrophils (10^9^/L)/the number of lymphocytes (10^9^/L). The PLR was calculated as the number of platelets (10^9^/L)/the number of lymphocytes (10^9^/L). The PNI was calculated as serum albumin (g/L) + 5*total lymphocyte count (10^9^/L). The SIRI was calculated as total neutrophil count (10^9^/L) *total monocyte count (10^9^/L)/total lymphocyte count (10^9^/L).

### SBRT

In our study, SBRT was performed with CyberKnife (Accuray Incorporated, Sunnyvale, CA, USA). All patients were implanted with gold fiducial tumor markers under ultrasound or CT guidance. Gold fiducials were placed in the lesion. For patients with poor performance status or tumors near large blood vessels, which were more at risk of multiple punctures, one gold fiducial was placed. Implantation of 3-5 fiducials was preferred. After implantation of the fiducials, a CT scan was performed to observe whether the markers were in the correct position and whether there were complications such as pneumoperitoneum or bleeding. In addition, patients were asked to stay in bed for 6 hours. The next day, CT scan was performed to observe whether the fiducials shifted and whether there were complications. Then, CT positioning scan was performed 7 days after the gold fiducials were implanted. Respiration synchronous tracking (Synchrony) was used to track the movement of the fiducials instead of tumor movements for simultaneous irradiation.

During abdominal CT simulation positioning, patient was in a supine position and fixed with a vacuum pad. Patient was asked to fast for at least 4 hours before positioning. To clearly show the gastrointestinal structures, 100-150 mL of contrast agent was taken orally 30, 20 and 10 minutes before the CT scan. At the same time, intravenous contrast was used to clearly show the lesions. The CT scans encompassed the entire circumference of the body contour with coverage from 15 cm above to 15 cm below the gold fiducial or the lesion. CT slice thickness was 1 mm. The gross tumor volume (GTV) was determined based on the tumor volume. Clinical tumor volume (CTV) was equal to GTV. GTV was expanded 0-5 mm to form the planning target volume (PTV). The PTV was modified by a radiation oncologist to avoid overlapping with the gastrointestinal tract. When the CyberKnife treatment plans were evaluated, the maximum single point dose was used as the limiting standard for serial organs and the maximum single dose of part volume was used as the limiting standard for parallel organs (14, 29). Because the median number of fractions was 5, organs at risk (OAR) dose constraints applied for five fraction SBRT was used in this study (**Supplementary Table 1**). SBRT was performed once a day, usually five days a week.

### Chemotherapy

The pancreatic cancer multidisciplinary committee of our hospital usually followed the guidelines of the National Comprehensive Cancer Network and decided the treatment plan based on the patient’s basic situation. The chemotherapy regimens were based on gemcitabine. The median number of chemotherapy cycles was 4 (rang of 0-7 cycles).

### Pain assessment

Patients were asked at baseline to identify a “target symptom” (pain), the primary abdominal complaint that they hoped SBRT would relieve. At each of the follow-up visits they were asked to describe the target symptom severity compared to baseline as either “worse”, “unchanged”, “improved” or “resolved.” The latter two options, “improved” or “resolved”, were regarded as “response.” Pain was scored using the visual analogue scale (VAS) and classified into four groups: painless group (score 0), mild pain group (score 1-3), moderate pain group (score 4-6), and severe pain group (score 7-10).

### Follow-up and statistics

During the treatment period, the patients underwent physical examination and laboratory tests every week, including blood routine, liver function, renal function, electrolytes, urine routine and stool routine. Follow-up was performed 1 month after the completion of SBRT and every 3 months thereafter, including physical examination, laboratory examination and imaging examination. Imaging examinations included chest CT, abdominal CT, and pelvic CT. If necessary, head MRI, bone scan, abdominal MRI, and PET-CT would be performed. The toxicity assessment was performed using the National Cancer Institute Common Terminology Criteria for Adverse Events version (CTCAE) 5.0. Acute toxicity was defined as events that occurs within 90 days from the start of radiotherapy. Late toxicity was defined as events that occurs more than 90 days after the start of SBRT.

Overall survival (OS) was defined as the time from the start of SBRT to the date of the last follow-up or death. Progression-free survival (PFS) was defined as the time from the start of SBRT to the date of progression at any site. Local failure (LF) referred to tumor recurrence within the irradiated volume, and was defined as the time from the start of SBRT to development of local failure or time to last known negative imaging. Death without the event of interest was a competitive event. Metastatic failure (MF) was defined as new metastases from the start of SBRT. Death without the event of interest was a competitive event. The cumulative incidence function was estimated using competitive risk analysis (Gray’s test) (30, 31). X-tile soft (32) was used to determine the optimal cut-off values for continuous variables. Kaplan-Meier method and Cox proportional hazard model were used for survival analysis. Only variables with P<0.05 from the univariate analyses were explored in the multivariable analysis. All statistical analyses were performed using SPSS 24.0 statistical software and R packages, and P<0.05 was statistically significant.

## Results

### Patient characteristics

A total of 22 locoregional recurrent pancreatic cancer patients after radical resection treated with SBRT were included into the study (**Table 1**). The patients ranged in age from 37 to 81 years (median age was 65 years). Among them, 12 were male (54.5%) and 10 were female (45.5%). The Eastern Cooperative Oncology Group performance status (ECOG) scores of patients were between 0 and 2. The main symptoms prior to SBRT were abdominal/back pain (n=16, 72.7%), epigastric discomfort (n=3, 13.6%), anorexia (n=3, 13.6%) and weight loss (n=2, 9.1%). The recurrent sites were mainly located in the remnant pancreas, around abdominal trunk and superior mesenteric artery.

**Table 1:** Patients’ characteristics.

Characteristics	No. of patients (%)
Patients	22 (100%)
Gender	
Male	12 (54.5%)
Female	10 (45.5%)
Age (years), median (range)	65 (37–81)
Performance status	
0	3 (13.6%)
1	13 (59.1%)
2	6 (27.3%)
Primary pancreatic tumor location	
Head	11 (50.0%)
Body/tail	11 (50.0%)
Type of surgery	
Radical resection	22 (100%)
Site of recurrence	
Residual pancreas	5 (22.7%)
Regional lymph nodes	17 (77.3%)
Systemic therapy	
Chemotherapy before SBRT	12 (54.5%)
Chemotherapy after SBRT	16 (72.7%)
Chemotherapy before and after SBRT	9 (40.9%)
No chemotherapy	3 (13.6%)
Chemotherapy cycles, median (range)	4 (0-7)
Chemotherapy regimens	
Gemcitabine monotherapy	2 (10.5%)
Gemcitabine and capecitabine	1 (5.3%)
Gemcitabine and S-1	11 (57.9%)
Gemcitabine and oxaliplatin	2 (10.5%)
Gemcitabine and nitolimumab	1 (5.3%)
Nab-paclitaxel and S-1	2 (10.5%)
Time to recurrences§ (months), median (range)	7.3 (3.4-30.6)
Time from recurrences to SBRT※ (months), median (range)	1.4 (0.2-10.9)
Pre-SBRT CA19–9 (U/ml), median (range)	240 (10–2200)
Pre-SBRT CA125 (U/ml), median (range)	14.5 (5-140)
Pre-SBRT CA242 (U/ml), median (range)	59 (2-285)
Pre-SBRT CEA (ng/ml), median (range)	4.5 (1-157)
SIRI, median (range)	0.628 (0.231-4.683)
BMI, median (range)	21.1 (17.3-26.4)
PLR, median (range)	136 (70.3-457)
NLR, median (range)	1.91 (0.62-8.15)
PNI, median (range)	47 (37-54)
LDH (U/L), median (range)	161 (120-259)

CA19-9, carbohydrate antigen 19–9; CEA, carcinoembryonic antigen; CA125, carbohydrate antigen 125; CA242, carbohydrate antigen 242; SIRI, systemic inflammation response index; NLR, neutrophil to lymphocyte ratio; PLR, platelet to lymphocyte ratio; PNI, prognostic nutritional index; LDH, lactate dehydrogenase; SBRT, stereotactic body radiotherapy.

§The time between curative surgery and initial diagnosis of recurrent disease.

※The time between initial diagnosis of recurrent disease and SBRT.

### Treatment characteristics

22 patients with a total of 24 tumor lesions were treated by SBRT. Among them, 2 patients received SBRT to 2 regional recurrent lymph nodes. The median time from the diagnosis of relapsed pancreatic cancer to SBRT was 40.5 days (range of 5-327 days). Among these patients, 12 patients underwent chemotherapy before SBRT and 16 patients underwent systemic chemotherapy after SBRT. The most common chemotherapy regimen was gemcitabine-based chemotherapy (**Table 1**).

The median GTV volume was 24.2cc (range of 9.2-122.4cc). The median PTV volume was 44.4cc (range of 15.7-150.4cc). The median percentage of PTV coverage was 73.1% (range of 57.3%- 94.7%). The treatment duration was 4-9 days. The median prescribed dose was 40 gray (Gy) (range of 30-50 Gy) and dose was given in 4 to 7 fractions. α/β was assumed to be 10 and median BED_10_ was 69.3 Gy (range of 48.0-100.0 Gy). The median prescription isodose was 75.5%. SBRT planning and delivery variables were summarized in **Table 2**.

**Table 2:** SBRT planning and delivery variables (N = 22 patients).

Variables	Median (range)
Prescription dose, Gy	40 (30.0-50.0) /4-7 fractions
Median BED_10_, Gy	69.3 (48.0-100.0) /4-7 fractions
Fraction dose (Gy per fraction)	7 (6.0-10.0)
Min dose to PTV (Gy)	21.7 (16.1-41.8)
Max dose to PTV (Gy)	50 (36.1-63.4)
PTV volume (cc)	44.4 (15.7-150.4)
PTV Coverage§ (%)	73.1 (57.3-94.7)
Number of beams	161.5 (88-240)
Prescription isodose line (%)	75.5 (67-83)
HI	1.33 (1.20-1.49)
CI	1.12 (1.06 -1.56)
nCI※	1.55 (1.15-2.28)
Target size (cm), median (range)	4.6 (2.9-9.6)

§Percentage of PTV volume was covered by prescription dose.

※The data of the CI multiplied by the ratio of the total tumor volume to the tumor volume receiving the prescription isodose or more.

BED, biological effective dose; Gy, gray; PTV, planning tumor volume; CI, conformity index; nCI, new conformity index; HI, homogeneity index; SBRT, stereotactic body radiotherapy.

### Survival analysis

#### Overall survival and progression free survival

**Figure 1:**
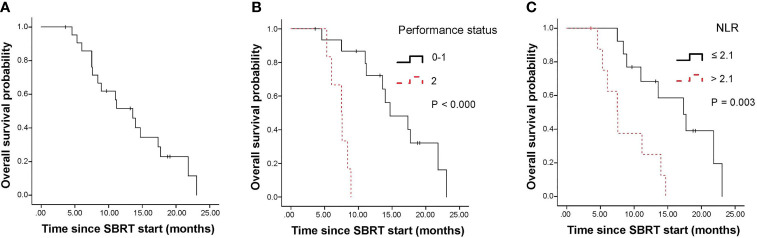
Kaplan-Meier curves for overall survival. Survival curves are displayed for the entire cohort **(A)** and stratified based on pre-SBRT performance status **(B)** and pre-SBRT NLR cutoffs **(C)**. SBRT, stereotactic body radiotherapy; NLR, neutrophil-to-lymphocyte ratio.

The median follow-up period for all 22 patients was 19.1 months (95% CI, 18.43-19.77 months). The median survival for surgery and recurrence were 23.07 months (95% CI, 20.67-25.47 months); and 15.1 months (95% CI, 10.77-19.43 months). The median overall survival (OS) of all patients from the start of SBRT was 13.6 months (95% CI, 9.6-17.5 months; **Figure 1A**). The 1-year OS rate of all patients was 51.6%. By univariate analysis, pre-SBRT CA19–9, pre-SBRT CEA, pre-SBRT CA242, pre-SBRT CA125, number of positive tumor markers before SBRT, PTV volumes, performance status, SIRI, NLR and PNI were found to be significantly associated with OS (**Table 3**). The multivariable analysis showed that 0-1 performance status (HR 12.10, 95% CI 2.04-71.81, P=0.006; **Figure 1B**) and ≤2.1 pre-SBRT NLR (HR 4.05, 95% CI 1.21-13.59, P=0.023; **Figure 1C**) were independent predictors of OS (**Table 4**).

**Table 3:** Univariate analysis of factors affecting PFS and OS.

Variables	Category	PFS		OS	
		Median PFS (95% CI) in months	*p* value	Median OS (95% CI) in months	*p* value
Gender	Male	7.5 (2.88-12.12)	0.765	11 (4.45-17.55)	0.514
	Female	6.9 (1.79-12.01)		13.97 (7.39-20.55)	
Age (years)	≤ 65	7.5 (3.72-11.28)	0.21	11.17 (5.60-16.74)	0.472
	> 65	10 (3.77-16.23)		13.57 (8.97-18.17)	
Tumor location	Head	5.87 (2.09-9.65)	0.372	11 (7.12-14.88)	0.330
	Body/tail	8.5 (6.39-10.61)		13.97 (12.98-14.96)	
Pre-SBRT CA19–9 (U/mL)	≤ 420	8.5 (5.11-11.90)	0.011	14.7 (9.16-20.24)	0.002
	> 420	3.6 (2.40-4.80)		7.57 (4.73-10.41)	
Pre-SBRT CEA (ng/ml)	≤ 10	8.5 (4.60-12.40)	0.04	13.97 (7.77-20.17)	0.007
	> 10	3.6 (0-8.16)		7.5 (4.66-10.35)	
Pre-SBRT CA242 (U/mL)	≤ 80	8.5 (3.73-13.27)	0.006	17.33 (11.94-22.72)	0.000
	> 80	4.1 (3.28-4.92)		7.57 (3.62-11.52)	
Pre-SBRT CA125 (U/mL)	≤ 35	10 (5.36-14.64)	0.009	14.7 (7.76-21.64)	0.008
	> 35	4 (3.14-4.86)		7.5 (4.34-10.66)	
Number of positive tumor markers before SBRT*	≤ 2	8.5 (5.36-11.64)	0.012	13.97 (11.97-15.97)	0.002
	> 2	3.6 (2.76-4.44)		6.03 (2.28-9.78)	
BED (Gy)	≤ 71.4	6.9 (4.13-9.67)	0.399	8.4 (0.25-16.55)	0.244
	> 71.4	7.6 (0-17.72)		13.97 (5.79-22.15)	
PTV volumes (cc)	≤ 72.8	8.5 (3.6-13.4)	0.063	14.7 (10.39-19.01)	0.002
	> 72.8	5.87 (2.18-9.56)		7.5 (2.84-12.16)	
Target size (cm)	≤ 5.3	8.5 (1.31-15.70)	0.452	13.97 (12.13-15.81)	0.06
	> 5.3	7.1 (5.14-9.06)		7.5 (4.64-10.36)	
Performance status	0-1	10 (6.75-13.25)	0.002	14.7 (9.03-20.37)	0.000
	2	4.1 (3.26-4.94)		7.5 (5.65-9.35)	
Time to recurrences (Months)	≤ 6.3	7.5 (6.33-8.67)	0.318	8.9 (4.81-12.99)	0.283
	> 6.3	10 (3.17-16.83)		13.97 (12.20-15.75)	
Site of recurrence	Residual pancreas	6.9 (0.89-12.91)	0.128	13.57 (6.29-20.85)	0.339
	Regional lymph nodes	8.5 (6.59-10.41)		11.17 (4.40-17.94)	
Chemotherapy cycles	≤ 4	7.6 (6.34-8.86)	0.357	13.57 (8.87-18.27)	0.622
	> 4	7.5 (0-16.23)		11.0 (2.02-19.98)	
SIRI	≤ 0.6	10.2 (2.32-18.08)	0.042	17.7 (7.83-27.57)	0.038
	> 0.6	7.1 (2.62-11.58)		8.9 (4.61-13.19)	
BMI	≤ 19.6	7.1 (2.06-12.14)	0.536	8.4 (6.72-10.08)	0.256
	> 19.6	7.6 (4.31-10.89)		13.97 (9.50-18.45)	
PLR	≤ 219.6	10 (4.78-15.22)	0.061	13.57 (2.95-24.19)	0.136
	> 219.6	5.87 (1.07-10.67)		11.17 (1.75-20.59)	
NLR	≤ 2.1	10 (2.84-17.164)	0.011	17.33 (11.12-23.54)	0.003
	> 2.1	4.3 (3.91-4.69)		7.5 (5.37-9.63)	
PNI	≤ 43.9	5.87 (2.24-9.504)	0.098	7.5 (5.15-9.85)	0.014
	> 43.9	8.5 (6.45-10.55)		13.97 (9.71-18.23)	
LDH	≤ 156	7.6 (7.31-7.89)	0.06	21.8 (10.73-32.87)	0.051
	> 156	7.1 (2.18-12.02)		8.9 (0.92-16.88)	

PFS, progression-free survival; OS, overall survival; BED, biological effective dose; Gy, gray; PTV, planning tumor volume; CA19-9, carbohydrate antigen 19–9; CEA, carcinoembryonic antigen; CA125, carbohydrate antigen 125; CA242, carbohydrate antigen 242; SIRI, systemic inflammation response index; NLR, neutrophil to lymphocyte ratio; PLR, platelet to lymphocyte ratio; PNI, prognostic nutritional index; LDH, lactate dehydrogenase; SBRT, stereotactic body radiotherapy.

*Number of positive tumor markers before SBRT, the tumor markers include CA19-9, CA242, CA125 and CEA.

**Table 4:** Multivariable analysis of PFS, OS and first events as MF after SBRT.

Variables	Category	PFS		OS		First events as MF	
		HR (95% CI)	*p*-value	HR (95% CI)	*p*-value	HR (95% CI)	*p*-value
Performance status	0-1	Ref.	0.006	Ref.	0.006	–	
	2	6.27 (1.68-23.43)		12.10 (2.04-71.81)		–	–
NLR	≤ 2.1	–	–	Ref.	0.023	–	–
	> 2.1	–	–	4.05 (1.21-13.59)		–	–
Pre-SBRT CA19–9 (U/mL)	≤ 420	–	–	–	–	Ref.	0.047
	> 420	–	–	–	–	9.19 (1.03-82.08)	

PFS, progression-free survival; OS, overall survival; MF, metastatic failure; NLR, neutrophil to lymphocyte ratio; SBRT, stereotactic body radiotherapy; CA19-9, carbohydrate antigen 19–9.

**Figure 2:**
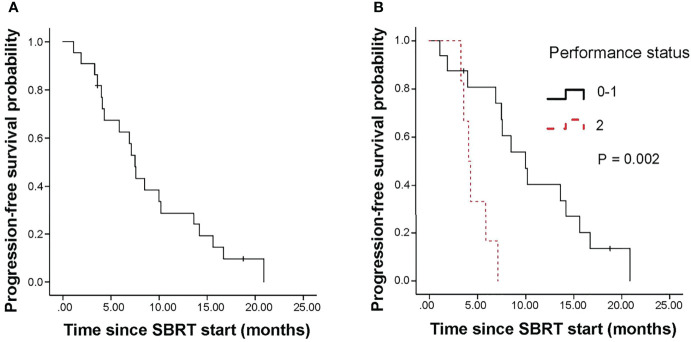
Kaplan-Meier curves for progression free survival. **(A)** shows of all patients; **(B)** shows pre-SBRT performance status. SBRT, stereotactic body radiotherapy.

The median progression-free survival (PFS) of all patients was 7.5 months (95% CI, 6.5-8.5 months; **Figure 2A**). The 1-year PFS rate of all patients was 28.9%. In univariate analysis, pre-SBRT CA19–9, pre-SBRT CEA, pre-SBRT CA242, pre-SBRT CA125, number of positive tumor markers before SBRT, performance status, SIRI and NLR were found to be significantly associated with PFS (**Table 3**). In multivariable analysis, only 0-1 performance status (HR 6.27, 95% CI 1.68-23.43, P=0.006; **Figure 2B**) was significantly associated with longer PFS (**Table 4**).

#### Local failure and metastatic failure

**Figure 3:**
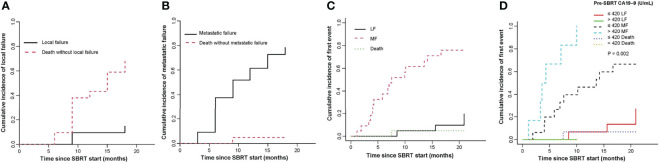
Cumulative incidence curves show the probability of local failure **(A)** and metastatic failure **(B)** and first events in the entire cohort **(C)** and first events according to pre-SBRT CA19–9 level **(D)**. SBRT, stereotactic body radiotherapy; LF, local failure; MF, metastatic failure; CA19-9, carbohydrate antigen 19-9.

5 patients experienced local failure (LF). 16 patients suffered metastatic failure (MF). 2 patients experienced simultaneous LF and MF. The median time to LF was 15.6 months. The 1-year cumulative incidence of LF was 9.5% (95%CI, 1.5-26.9%; **Figure 3A**).

The median time to MF for all patients was 6.4 months. The cumulative incidence of MF at 1 year was 61.5% (95%CI, 36.46-79.11%, **Figure 3B**). In univariate analysis, pre-SBRT CA19-9 (P=0.002), pre-SBRT CA242 (P=0.001), number of positive tumor markers before SBRT (P=0.003), PTV (P=0.018), performance status (P=0.003), site of recurrence (P=0.022), SIRI (P=0.049), NLR (P=0.003), PNI (P=0.014) and LDH (P=0.043) were significant factors for MF (**Supplementary Table 2**). In multivariable analysis, there were no significant factors for MF.

For the entire cohort, failures were more likely to occur outside the irradiation field than inside the field. The rate of metastatic failure as a first event was higher than that of first event of local failure (**Figure 3C**). By univariate analysis, pre-SBRT CA19-9 (P=0.002), pre-SBRT CA242 (P=0.001), number of positive tumor markers before SBRT (P=0.003), PTV (P=0.018), performance status (P=0.003), site of recurrence (P=0.022), NLR (P=0.003) and PNI (P=0.014) were associated with first event metastatic failure (**Supplementary Table 2**). By multivariable analysis, only pre-SBRT CA19-9 (HR 9.19, 95% CI 1.03-82.08, P=0.047; **Figure 3D**) was significant for first event metastatic failure (**Table 4**).

#### Pain relief

Prior to radiotherapy, 16 patients had abdominal and/or back pain. There were 5 cases of mild pain, 8 cases of moderate pain, and 3 cases of severe pain. Of the 16 patients for pain severity evaluation at 12 weeks after SBRT, 8 had complete cessation of pain, 8 had pain relief. The relief or complete cessation of the target pain was achieved in 43.75% of the available patients at the end of SBRT, 100% after 6 weeks, and 100% after 12 weeks (**Supplementary Figure 1**). The median time to pain relief after SBRT was 13 days (0-30 days). The median VAS scores were 5 (range: 2-8) before SBRT. At 6 weeks after SBRT, the median VAS score decreased significantly to 1.5 (range: 0-4) (P<0.000). The pain relief or complete cessation of pain with analgesic drugs was 100%.

#### Toxicity

Overall, the treatment was well tolerated. All patients received complete SBRT. There were no therapy-related deaths. In terms of acute toxicity, grade 1 including fatigue (6, 27.3%), anorexia (6, 27.3%) and nausea (4, 18.2%) was often observed. The incidence of non-hematological toxicity no higher than grade 2 was 45.5% (10/22). For overall hematological toxicity, cases of grades 2 and 3 accounted for 22.7% (5/22) and 4.5% (1/22) of total cases, respectively. No acute toxicity of grade 4 or 5 was observed. In terms of late toxicity, no treatment-related toxicity was found during follow-up. The observed SBRT-related toxicity events were summarized in **Table 5**

**Table 5:** Acute and late toxicities (N = 22 patients).

Toxicity	Grade 1 (%)	Grade 2 (%)	Grade 3 (%)	Grade 4 (%)	Grade 5 (%)
Acute					
Hematological					
Leukopenia	4 (18.2)	3 (13.6)	0 (0)	0 (0)	0 (0)
Neutropenia	1 (4.5)	1 (4.5)	0 (0)	0 (0)	0 (0)
Anemia	3 (13.6)	1 (4.5)	0 (0)	0 (0)	0 (0)
Thrombocytopenia	2 (9.1)	0 (0)	1 (4.5)	0 (0)	0 (0)
Non-hematological					
Hyperbilirubinemia	0 (0)	0 (0)	0 (0)	0 (0)	0 (0)
Elevated ALT	0 (0)	0 (0)	0 (0)	0 (0)	0 (0)
Elevated AST	0 (0)	0 (0)	0 (0)	0 (0)	0 (0)
Fatigue	6 (27.3)	1 (4.5)	0 (0)	0 (0)	0 (0)
Anorexia	6 (27.3)	1 (4.5)	0 (0)	0 (0)	0 (0)
Diarrhea	2 (9.1)	0 (0)	0 (0)	0 (0)	0 (0)
Constipation	1 (4.5)	0 (0)	0 (0)	0 (0)	0 (0)
Nausea	4 (18.2)	2 (9.1)	0 (0)	0 (0)	0 (0)
Vomiting	2 (9.1)	0 (0)	0 (0)	0 (0)	0 (0)
Pain	1 (4.5)	0 (0)	0 (0)	0 (0)	0 (0)
Jaundice	0 (0)	0 (0)	0 (0)	0 (0)	0 (0)
Late					
Pain	0 (0)	0 (0)	0 (0)	0 (0)	0 (0)
GI Bleeding	0 (0)	0 (0)	0 (0)	0 (0)	0 (0)
Duodenal Stricture	0 (0)	0 (0)	0 (0)	0 (0)	0 (0)
Small bowel perforation	0 (0)	0 (0)	0 (0)	0 (0)	0 (0)
Stomach ulcer	0 (0)	0 (0)	0 (0)	0 (0)	0 (0)
Duodenal ulcer	0 (0)	0 (0)	0 (0)	0 (0)	0 (0)

ALT, alanine transaminase; AST, aspartate aminotransferase; GI, gastrointestinal.

## Discussion

Pancreatic cancer is characterized by high recurrence after operation. Although great progress has been made in comprehensive treatment (including surgery, chemotherapy, radiation therapy and immunotherapy) of pancreatic cancer, its prognosis is still poor (6, 33). Recurrent tumors after surgery can cause severe local symptoms. Active local treatment is thus required. Therefore, we conducted this retrospective study to investigate the role of SBRT in locoregional recurrent pancreatic cancer after operation. In the present study, we found that SBRT offered favorable survival, local tumor control and pain relief for locoregional recurrent pancreatic cancer patients.

Zhu et al. (34) recruited patients with postoperative locally recurrent pancreatic cancer who were randomly assigned to receive SBRT plus pembrolizumab and trametinib or SBRT plus gemcitabine. The median OS were 14.9 months and 12·8 months, respectively. This survival rate was comparable to our study. The median OS in this paper was 13.6 months.

The serum inflammation-based and/or nutritional markers have been reported to be associated with survival in various solid tumors, including pancreatic cancer (35–38) Mei et al. (35) retrospectively reviewed 66 studies with a total of 24,536 patients with advanced cancer. They found that increased pretreatment blood NLR may be correlated with worse survival. Goldstein et al. (37) reported that the median OS was significantly longer for nab-paclitaxel plus gemcitabine than that for gemcitabine alone in metastatic pancreatic cancer. Through further subgroup analysis, they found that CA19-9 level and NLR at baseline were independent predictive markers for OS. In our study, we determined that poor performance status and high NLR before SBRT were independent risk factors for poor prognosis by multivariable analysis. Therefore, potential mechanisms underlying the association between high NLR and worse prognosis should be investigated to identify strategies for the treatment of patients with pancreatic cancer. The possible mechanism is that a higher proportion of neutrophils was associated with an inflammatory response that suppresses the immune system by inactivating lymphocytes, activating T cells, and natural killer cells (39). Neutrophils can secrete tumor promoting factors such as TGF-β, IL-6 and IL-8, creating a stimulatory environment for tumor growth (40). In addition, neutrophils in the peripheral blood were shown to promote the efficiency of distant metastasis by interacting with circulating tumor cells and enhancing their metastatic phenotype through supporting cell cycle progression and accelerating metastasis seeding (41–43). It is well known that lymphocytes have antitumor properties and their depletion promote tumor progression (44). Therefore, the combination of high neutrophils and low lymphocytes as reflected by NLR values would represent ideal immune conditions to facilitate tumor progression and metastasis. Drugs that may potentially recruit, activate, inhibit or otherwise modulate the phenotypes of neutrophils in the tumor microenvironment are currently being studied in patients with cancer (20, 45).

In addition, the performance status may have impact on the individualized therapy, because good patient fitness can withstand intensive combination therapy and have a good quality of life. Many studies found that elevated NLR was associated with malnutrition, weight loss, cancer cachexia, substantial disease burden and poor performance status (46–49). Therefore, NLR should be considered as a further stratification factor in clinical trials to determine at diagnosis whether patients with high NLR should receive intensive treatment or only palliative treatment.

CA19-9 has been identified as the best tumor marker for the prognosis of pancreatic cancer (50–52). In addition, patients with two or three markers positive expression of CEA, CA19-9 and CA242 simultaneously had a shorter survival time (53). We tested multiple tumor markers, including CA199, CA242, CA125 and CEA. In the univariate analysis, we found that CA19-9 > 420U/mL, CA242 > 80U/ml, CA125 > 35 U/ml, CEA > 10 ng/ml and three or more positive tumor markers (CA19-9, CA242, CA125 and CEA) were independent indicators of worse prognosis of OS. Therefore, we suggest that patients with elevated levels of several serum tumor markers require urgent intensified treatment. These markers may help identify potential recurrent pancreatic cancer candidates who may benefit from SBRT.

Krishnan et al. found that a BED_10_ value greater than 70 Gy was associated with improved OS in pancreatic cancer (54). Zhu et al, found that a BED_10_ over 60 Gy can improve survival (55, 56). However, there was no survival benefits with a BED_10_ value higher than 71.4Gy in this paper. This is because most of lesions (50%) were treated with a BED_10_ over 70 Gy. Hence, for patients with high tumor markers and high NLR, escalated radiation doses may not be necessary, but a combination of multiple treatment modalities may be required.

It is reasonable to assess the local control of recurrent lesions. Comito et al. (57) analyzed the application of SBRT for patients with isolated local recurrence of R0 resected pancreatic cancer, and the median OS was 18 months. The 1 and 2-years local control rates were 91% and 82%, respectively. Zeng et al. (58) evaluated the efficacy and safety of SBRT in the treatment of patients with recurrent pancreatic adenocarcinoma at the abdominal lymph node or stump after surgery. They found that the median OS from the start of SBRT was 12.2 months and the 6-, 12-, and 24-month actuarial local control rates were 95.2%, 83.8%, and 62.1%, respectively. In this paper, the 1-year cumulative incidence of LF after SBRT was 9.5%. This is consistent with existing studies. This suggests that SBRT may achieve promising local tumor control in patients with locally recurrent pancreatic cancer. These results could be biased by the observed short OS, because patients may not have reached the endpoint of local tumor progression. As for patterns of failure, the first failure for all patients was more inclined to occur outside the irradiation area than in the irradiated area. In this paper, the multivariable analysis demonstrated that pre-SBRT CA19-9 was statistically significant for MF as a first event. There were high rates of metastatic failure occurred early in this study. Therefore, it is necessary to combine SBRT with additional systemic therapy.

For pancreatic cancer, reducing symptoms such as abdominal and/or back pain is considered to be the main goal of improving patients’ quality of life. A phase 2 multi-institutional study reported a significant improvement in pancreatic pain 4 weeks after SBRT (17) These patients with locally advanced pancreatic cancer received SBRT combined with gemcitabine. Two small retrospective studies have demonstrated that pain relief rates were 73-80% in elderly or medically inoperable pancreatic cancer patients after SBRT (59, 60). In addition, a systematic review reported an overall pain relief rate was 84.9% after SBRT in patients with locally advanced pancreatic cancer (61) Zeng et al. (58) found that symptom alleviation rate was 78.6% within a median of 8 days after SBRT. Our study found that 100% of patients experienced significant pain relief after SBRT. For the 16 patients who experienced pain before radiotherapy, the pain VAS score was significantly reduced after treatment. Because SBRT has a significant pain-relieving effect, it can reduce the use of analgesics, thereby improving the patients’ quality of life.

In developing a treatment plan for patients, SBRT toxicity is our first consideration. In this study, most patients experienced CTCAE grade 1-2 acute toxic events, and most of these symptoms were transient and resolved with conservative management. No late toxicity was reported, although the relatively short median OS may have underestimated the rate of late toxicities following SBRT. This study indicated that patients showed good tolerance to SBRT.

However, this study has the following limitations. (1) The number of patients used for analysis is small and it is a retrospective study. (2) The treatments (especially chemotherapy) vary with patients. (3) There were no objective indicators to assess the patients’ quality of life after receiving SBRT. (4) This study was performed at a single-center. (5) Our study included only patients that underwent SBRT, without a valid comparator. Future prospective studies should include more patients, a control group and objective evaluation indicators to clarify the specific benefits of SBRT.

In conclusion, SBRT is an alternative local treatment for locoregional recurrent pancreatic cancer after surgery that can achieve promising LC rates, significantly improve local symptoms such as pain, and is well tolerated without serious toxicities.

## Data availability statement

The raw data supporting the conclusions of this article will be made available by the authors, without undue reservation.

## Ethics statement

The studies involving human participants were reviewed and approved by ethics Committee of Jinling Hospital. The patients/participants provided their written informed consent to participate in this study.

## Author contributions

XS designed the study. XJ, BZ, and JW collected the data. XJ and WJ: manuscript drafting. WD, JH, and YL analyzed and interpreted the data. All the authors read and approved the final manuscript.

## Funding

The work is supported by grants from the Hospital Research Foundation of Jinling Hospital (YYQN2021084).

## Conflict of interest

The authors declare that the research was conducted in the absence of any commercial or financial relationships that could be construed as a potential conflict of interest.

## Publisher’s note

All claims expressed in this article are solely those of the authors and do not necessarily represent those of their affiliated organizations, or those of the publisher, the editors and the reviewers. Any product that may be evaluated in this article, or claim that may be made by its manufacturer, is not guaranteed or endorsed by the publisher.

## Supplementary material

Supplementary Figure 1 Abdomen/back pain severity over time. Abbreviations: EoSBRT, end of stereotactic body radiotherapy; w, week.

## Abbreviations

SBRT, stereotactic body radiotherapy; OS, overall survival; LF, local failure; MF, metastatic failure; SIRI, systemic inflammation response index; NLR, neutrophil-to-lymphocyte ratio; PLR, platelet-to-lymphocyte ratio; PNI, prognostic nutritional index; LDH, lactate dehydrogenase; CA19-9, carbohydrate antigen 19-9; CEA, carcinoembryonic antigen; CA125, carbohydrate antigen 125; CA242, carbohydrate antigen 242; PTV, planning tumor volume; BED, biological effective dose; CT, computed tomography; MRI, magnetic resonance imaging; 18F-FDG-PET/CT, 18-fluorodeoxyglucose positron emission tomography/computed tomography; GTV, gross tumor volume; CTV, clinical tumor volume; OAR, organs at risk; Gy, gray; VAS, visual analogue scale.
