# Supplementary figures and images for: Efficacy of stereotactic body radiation therapy for locoregional recurrent pancreatic cancer after radical resection
Source: Front Oncol. 2022 Jul 22;12:925043. doi: 10.3389/fonc.2022.925043 (PMC9353056; doi:10.3389/fonc.2022.925043)

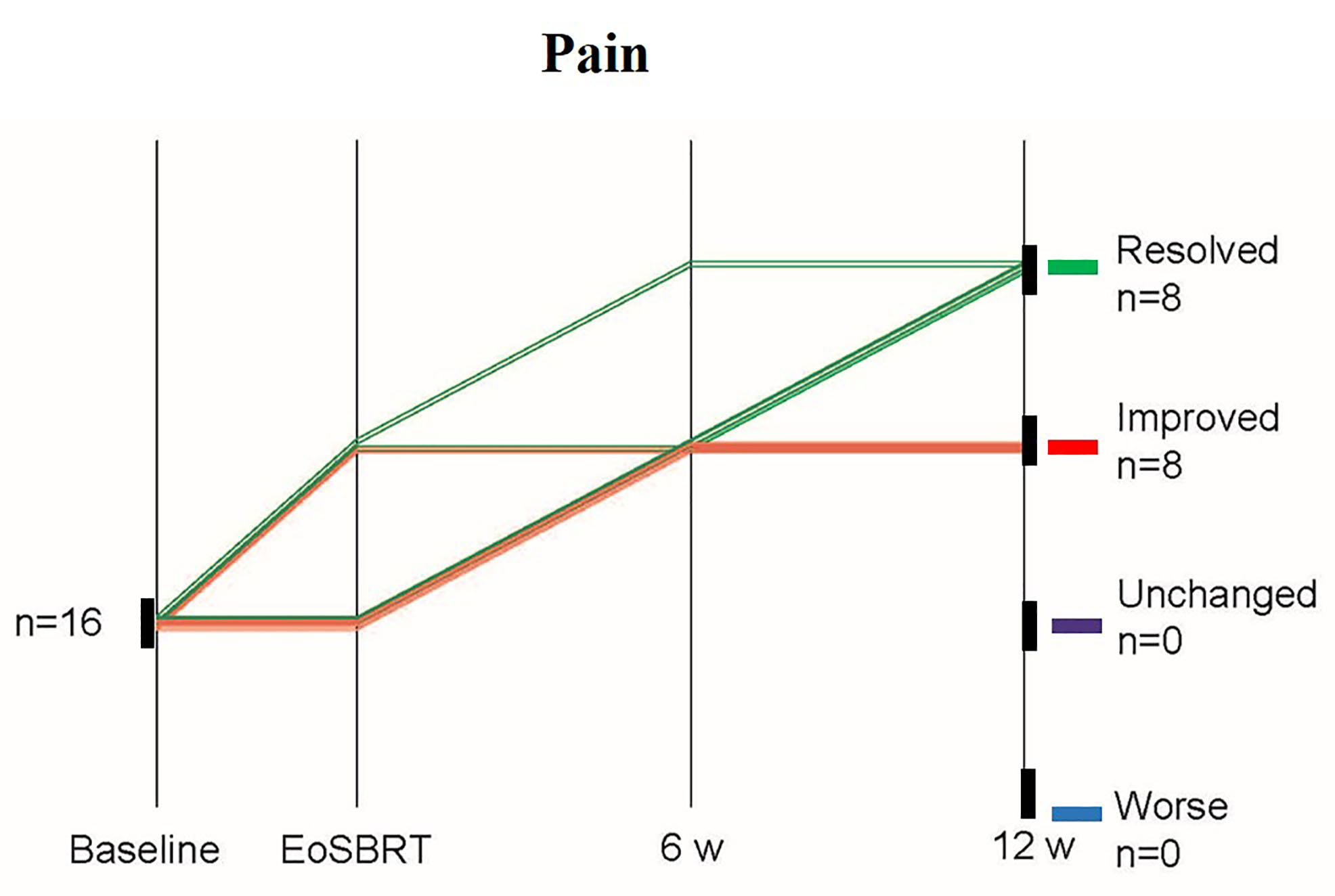

Supplement: Supplementary Figure 1 — Abdomen/back pain severity over time. Abbreviations: EoSBRT, end of stereotactic body radiotherapy; w, week. [file Image_1.jpeg]
