# Supplementary material for: Efficacy of stereotactic body radiation therapy for locoregional recurrent pancreatic cancer after radical resection
Source: Front Oncol. 2022 Jul 22;12:925043. doi: 10.3389/fonc.2022.925043 (PMC9353056; doi:10.3389/fonc.2022.925043)
Supplement: Supplementary file 2 [file Table_1.docx]

| **Supplementary table 1** OAR dose constraints applied for five fraction SBRT in this study | | | | | | | | | |
| --- | --- | --- | --- | --- | --- | --- | --- | --- | --- |
|  | D0.035 cc | D0.2 cc | D0.35 cc | D0.5 cc | D5.0 cc | D10.0 cc | D20 cc | V15Gy | Vspare |
| Duodenum | ≤30.0 Gy | — | — | — | 18 | 12.5 | — | — | — |
| Stomach | ≤30.0 Gy | — | — | — | — | 18 | — | — | — |
| Esophagus | ≤35.0 Gy |  |  |  | 19.5 |  |  |  |  |
| Small Bowel | ≤32.5 Gy | — | — | — | 19.5 | — | — | — | — |
| Colon | ≤35.0 Gy | — | — | — | — | — | 25 | — | — |
| Liver | — | — | — | — | — | — | — | — | Vtot - V21 Gy >700 cc* |
| Spinal cord | 25 | — | 20 | — | — | — | — | — | — |
| Left kidney | — | — | — | — | — | — | — | <35% | — |
| Right kidney | — | — | — | — | — | — | — | <35% | — |
| *The total volume of the liver minus the volume exposed to 21Gy was greater than 700cc. *Abbreviations:* OAR, organs at risk. | | | | | | | | | |
