# Supplementary material for: Efficacy of stereotactic body radiation therapy for locoregional recurrent pancreatic cancer after radical resection
Source: Front Oncol. 2022 Jul 22;12:925043. doi: 10.3389/fonc.2022.925043 (PMC9353056; doi:10.3389/fonc.2022.925043)
Supplement: Supplementary file 3 [file Table_2.docx]

| **Supplementary table 2. Univariate analysis of metastatic failure and first events after SBRT.** | | | |
| --- | --- | --- | --- |
| **Co-variates** | **Category** | **Metastatic failure** | **First events** |
|  |  | P-value | P-value |
| Gender | Female | 0.97 | 0.92 |
|  | Male |  |  |
| Age | ≤ 65 | 0.98 | 0.91 |
|  | > 65 |  |  |
| Primary location | Head | 0.16 | 0.13 |
|  | Body/tail |  |  |
| Pre-SBRT CA19–9 (U/mL) | ≤ 420 | 0.002 | 0.002 |
|  | > 420 |  |  |
| Pre-SBRT CEA (ng/ml) | ≤ 10 | 0.32 | 0.32 |
|  | > 10 |  |  |
| Pre-SBRT CA242 (U/mL) | ≤ 80 | 0.001 | 0.001 |
|  | > 80 |  |  |
| Pre-SBRT CA125 (U/mL) | ≤ 35 | 0.38 | 0.38 |
|  | > 35 |  |  |
| Number of positive tumor markers before SBRT* | ≤ 2 | 0.003 | 0.003 |
|  | > 2 |  |  |
| BED (Gy) | ≤ 71.4 | 0.75 | 0.87 |
|  | > 71.4 |  |  |
| PTV volumes (cc) | ≤ 72.8 | 0.018 | 0.018 |
|  | > 72.8 |  |  |
| Target size (cm) | ≤ 5.3 | 0.94 | 0.91 |
|  | > 5.3 |  |  |
| Performance status | 0-1 | 0.003 | 0.003 |
|  | 2 |  |  |
| Time to recurrences (Months) | ≤ 6.3 | 0.66 | 0.56 |
|  | > 6.3 |  |  |
| Site of recurrence | Residual pancreas | 0.022 | 0.022 |
|  | Regional lymph nodes |  |  |
| Chemotherapy cycles | ≤ 4 | 0.54 | 0.45 |
|  | > 4 |  |  |
| SIRI | ≤ 0.6 | 0.049 | 0.11 |
|  | > 0.6 |  |  |
| BMI | ≤ 19.6 | 0.78 | 0.73 |
|  | > 19.6 |  |  |
| PLR | ≤ 219.6 | 0.093 | 0.23 |
|  | > 219.6 |  |  |
| NLR | ≤ 2.1 | 0.003 | 0.003 |
|  | > 2.1 |  |  |
| PNI | ≤ 43.9 | 0.014 | 0.014 |
|  | > 43.9 |  |  |
| LDH | ≤ 156 | 0.043 | 0.071 |
|  | > 156 |  |  |
| *Abbreviations:* SBRT, stereotactic body radiotherapy; BED, biological effective dose; Gy, gray; PTV, planning tumor volume; CA19-9, carbohydrate antigen 19–9; CEA, carcinoembryonic antigen; CA125, carbohydrate antigen 125; CA242, carbohydrate antigen 242; SIRI, systemic inflammation response index; NLR, neutrophil to lymphocyte ratio; PLR, platelet to lymphocyte ratio; PNI, prognostic nutritional index; LDH, lactate dehydrogenase; SBRT, stereotactic body radiotherapy.  *Number of positive tumor markers before SBRT, the tumor markers include CA19-9, CA242, CA125 and CEA. | | | |
